# Factors associated with COVID-19 vaccine uptake among health professionals in Debre Markos town public health facilities, Northwest Ethiopia

**DOI:** 10.1371/journal.pgph.0002893

**Published:** 2024-04-03

**Authors:** Michu Belay, Tilahun Degu Tsega, Muluye Molla, Muluken Teshome

**Affiliations:** 1 Dejen Primary Hospital, Amhara regional Health Bureau, Dejen, Ethiopia; 2 Department of Public Health, College of Medicine and Health Sciences, Injibara University, Injibara, Ethiopia; 3 Department of Public Health, College of Health Sciences, Debre Markos University, Debre Markos, Ethiopia; Technical University of Kenya, KENYA

## Abstract

Globally, the COVID-19 pandemic has significantly increased morbidity and mortality. Health professionals are at the frontline of COVID-19 pandemic exposure and are identified as a priority target group that needs to receive COVID-19 vaccines. Data on Ethiopian healthcare workers’ use of the COVID-19 vaccine and associated factors, however, are scarce. Using a simple random sampling method, 398 health professionals were recruited through an institutional-based cross-sectional study design. Health professionals working in Debre Markos town public health facilities filled out a self-administered questionnaire that had been pretested to obtain the data. Then, the data were entered into Epi data version 4.2; and analyzed by SPSS 25. Descriptive statistics and multivariable logistic regression analysis were computed after model assumptions were checked. The adjusted odds ratio with 95% CI was calculated and statistical significance was declared at P-value < 0.05 after model adequacy was checked using the Hosmer-Lemeshow test. The uptake of the COVID-19 vaccine among health professionals was 61.56% (95% CI: 56.67%, 66.23%). Factors associated with the uptake of the COVID-19 vaccine were age > = 35 years (AOR: 4.39, 95% CI: 1.89, 10.19), having a higher income (>9056 Birr) (AOR: 1.79, 95% CI: 1.03, 3.10), who practiced COVID-19 Prevention methods (AOR: 2.39, 95% CI: 1.51, 3.77), Adulthood previous other immunization histories (AOR: 1.63, 95% CI: 1.15, 2.56) and having a chronic disease (AOR:1.90,95% CI: 1.07, 3.74). This study revealed that the uptake of the COVID-19 vaccine was low. Age > = 35 years, having a higher income, who practiced COVID-19 prevention methods, having adulthood previous immunization histories, and having chronic disease were statistically significantly identified factors for COVID-19 vaccine uptake. Therefore, policymakers and health managers should think about the requirement of immunization of healthcare workers and develop plans for administering the COVID-19 vaccine.

## Introduction

The World Health Organization (WHO) and other healthcare institutions have strove to produce vaccines and apply other forms of prevention, diagnosis, and treatment options in response to the COVID-19 pandemic [1, 2]. Even though coronavirus disease (COVID-19) control through vaccination depends on more than only vaccine efficacy and safety, the vaccine needs to be used by health professionals and the general public as well for effective control [3, 4].

Globally, coronavirus is a serious public health issue that affects people of all ages, races, and socioeconomic levels, particularly health professionals and patients with co-morbid conditions [5]. COVID-19’s massive global destruction has had an impact on almost every element of existence, which caused significant morbidity and mortality of human beings, and major social, educational, and economic losses throughout the world [1, 5].

Nearly 13.5 billion vaccine doses had been given by the end of July 2023 [6]. About 66% of the world’s population had got the vaccination in at least one dose; and 32% of the population took booster dose [7]. However, there are significant disparities in the pace of vaccine use around the globe, with just three nations, the United States of America, China, and India, administering more than 60% of all vaccination doses given [6–8]. Still, only 29% percent of dosages were administered in low-income nations in contrast to 89% in high and upper-middle-income nations [6, 7, 9].

As with the general population, the COVID-19 vaccine uptake among health professionals varies from country to country [9]. The disparity of taking the vaccine was high between high and low-income countries [9, 10]. For instance, in the U.S.A, the overall health professionals vaccine uptake was 78.2% and 78.6% [11, 12], but 92% among active workers in Health facilities [13]; in Los Angeles, California 96.0% received the vaccine [14]; and in Canada also 82% had got the vaccination with at least one dose of a COVID-19 vaccine, and out of the total 57% were fully vaccinated [15]. Likewise, in Saudi Arabian countries, one study reported 33.27% of health professionals were vaccinated [16] and another study in Saudi Arabia stated that 89.02% health care professionals took the vaccine at least once [17]. In China, 66.5% were also vaccinated with at least one dose of the vaccine, whereas 34.9% were fully vaccinated [18, 19]; in England, 64.5% and 89% [20, 21], in Greece from 33.3% to 94.5% [22] took COVID-19 vaccine. In the European countries, a high proportion of the Italian health professionals (98.9%) took the vaccine in opposite to the Cyprus and Germany health professionals (30%) [10].

A review done in Africa showed that the overall health professionals’ vaccine acceptance rate was 48%, whereas in East Africa it is 49% [23]. In Somalia, the vaccine was taken by 48.6% of Health care providers. Among these, thirty-eight percent of them took the vaccine fully [24]. In Egypt, around 28% of health care workers accepted to take the COVID-19 vaccine [25]. In Ethiopia, the overall COVID-19 vaccine acceptance rate among health professionals was 54.59% [26] but, an online study showed that 62.1% of healthcare professionals were vaccinated with at least one dose of the COVID-19 vaccine [27]. Another study in Addis Ababa, Ethiopia, revealed that 75.8% of health care professionals took at least one dose of COVID-19 vaccine [28].

The uptake of the COVID-19 vaccine among health professionals was affected by factors such as sex, age, level of awareness [16, 20, 29], ethnicity, occupation, previous history of infection [21], better socioeconomic status, confidence level of self-perceived COVID-19 risk, having COVID-19 vaccines’ information, chronic illness, religious reasons, concerns about the safety and effectiveness of vaccines, medication, pregnancy, fertility, breastfeeding, ethical reasons, previous COVID-19 diagnosis, self-estimation that COVID-19 is not a severe disease, residence, educational status, monthly income, place of work, type of profession, staffing, perception of own and their family member health status, tested for COVID-19, history of receiving another vaccine, involved in COVID-19 isolation center, and history of contact with confirmed COVID-19 patients [16, 18, 20–22, 27, 29].

The COVID-19 situation in Ethiopia has caused a significant socioeconomic crisis. The government of Ethiopia has been acting in several ways to decrease the consequences of the disease and to stop its spreading [30]. The Federal Ministry of Health (FMOH) and Ethiopian Public Health Institute (EPHI) in collaboration with partners have intensified response efforts to prevent the spread and severity of COVID-19 disease in Ethiopia through the activated national and regional Public Health Emergency Operations Centre (PHEOC). Besides, the laboratory diagnosis capacity has been expanded to other national institutions, sub-national, and private laboratories [31].

Health professionals are at high risk of COVID-19 infection, contributing to further spread because health professionals have many contacts with other populations in their work and living areas [31]. Health Care Workers are among the first group to receive the vaccine. Uptake of the COVID-19 vaccine among healthcare workers is mandatory to minimize and reduce the chain of transmission of the disease. The acceptance and use of the COVID-19 vaccine is an important challenge to be addressed. The attitudes of healthcare workers towards COVID-19 vaccines and their uptake could affect the communities’ uptake of vaccines against COVID-19. However, the uptake of the COVID-19 vaccine still matters among health professionals [3, 31] and the uptake varies between regions and sub-regions of a continent and country. Hence, there is limited data about the utilization of the COVID-19 vaccine among health professionals in Ethiopia, particularly in the study area. So this study aimed to determine the uptake of COVID-19 vaccine and associated factors among health professionals in Debre Markos town public health facility.

## Methods

### Ethics statement

The study protocol was reviewed and approved by the ethical review committee of Debre Markos University, College of Health Sciences **(Ref. N****o**: **HSC/R/C/ser/CO/65/11/14)** and informed consent was obtained from each study participant. Permission letters were also obtained from each health facility. Names of participants and other personal identifiers were not included in the data collection tool. Confidentiality of information was kept properly.

### Study settings

An institution-based cross-sectional study design was used. This study was conducted from January to February 2022, at Debre Markos town public health facility in East Gojjam zone, Northwest Ethiopia. Debre Markos administrative town is among one of the 19 districts and 2 administrative towns in the East Gojjam zone. Debre Markos is the capital city of the East Gojjam Zone, which is found about 299 km northwest of Addis Ababa, the capital city of Ethiopia, and 265 km southeast of Bahir Dar, the capital city of the Amhara region. Currently, Debre Markos town has 1 public hospital and 3 public health centers. In these public health facilities, a total of 707 healthcare professionals are working [32].

### Population

All health professionals who have worked at the public health facilities of Debre Markos town during the data collection period were the study population. Health professionals who were on maternal, annual, and sick leave for more than one month during the data collection period were excluded from this study.

### Sample size and sampling procedure

The sampling size for this study was determined using a single population proportion formula by taking the following assumptions: 95% confidence interval (Z_α/2_ = 1.96), the margin of error (d) 5%, 10% non-response rate, and 62.1% uptake of COVID-19 vaccine [27]. Then, the final minimum sample size required for this study was 398.

There were 707 health professionals in the Debre Markos town public health facilities, one comprehensive specialized hospital, and three health centers [32]. A list of all health professionals for each public health facility was framed using their ID from each health facility’s human resource department, and then the sampled health professionals were drawn through a simple random sampling technique/Computer-generated random number/ proportionally allocating the sample to each public health facility. If the selected professional was not present during the data collection time, they went back again until they got the selected professional. If the selected professional was not present until the 3^rd^ week of data collection time, they were considered as a non-response participant.

### Variables

#### Dependent variable

Uptake of Covid-19 vaccine.

#### Independent variables

**Socio-demographic and economic factors**: age, sex, religion, ethnicity, marital status, partner occupation, family size, years of experience, average monthly income, and type of profession.
**COVID-19-related factors**: previous COVID-19 infection, history of contact with COVID-19, patients, knowledge about COVID-19, attitude towards COVID-19 vaccine, source of information about COVID-19 & the vaccine, and practicing COVID-19 preventive measures.
**Health-related factors**: type of health institution, previous exposure to adulthood immunization, availability of COVID-19 vaccine, working area in the facility, and chronic illness status.

### Operational definitions

**Uptake of vaccine**- is the proportion of eligible health professionals who received a vaccine during a specific period [33]. In this study, we had got the vaccination with at least one dose of the COVID-19 vaccine was considered as uptake [27].

**Good knowledge of COVID-19**- when study participants respond with the right answer to 75% or more of the knowledge questions [31].

**Favorable attitude to COVID-19 vaccine**: when study participants respond positively to 50% or more of attitude questions [3].

**Practicing COVID-19 preventive measures**: when the study participants wear the mask and wash their hands with soap and/or sanitizer at the appropriate time and place.

### Data collection tools and procedures

Questionnaires were prepared in the English language from different works of literature. Data was collected by using a structured self-administered questionnaire. Four health professionals who have good communication skills were recruited for data collection. One health professional who had experience in data collection supervised the data collection process in addition to the principal investigator.

### Data quality assurance

For the data collection instrument (self-administer questionnaire), a pretest was done on 5% of study participants that wrought at Dejen town public health facilities. Based on the results of the pretest, grammatical modifications to the questionnaire were done. One day of orientation was given to the data collectors and supervisor on the objectives, procedures, and data collection techniques of the study. The collected data were checked for their completeness by all data collectors and a supervisor. After the completeness was checked, the data was entered into Epi data version 4.2.

### Data processing and analysis

The collected data from each participant were entered into Epi-Data version 4.2 and exported into SPSS version 25 for further cleaning, coding, and analysis. First, a descriptive analysis was performed and summary measures were displayed using text narrations, tables, and graphs.

Variables that showed significance at a P-value of 0.2 during bi-variable logistic regression analysis were entered into multivariable logistic regression analysis. The variables with P-value <0.05 in the multivariable regression model were interpreted after the fitness have been checked. The overall model adequacy of the multivariable logistic regression was checked using Hosmer-Lemeshow goodness-of-fit test (P-value = 0.968) and classification table (Correctly classified = 78.26%).

Lastly, variables having p values < 0.05 were accepted as statistically significant factors of the uptake of the COVID-19 vaccine, and final model interpretation and reporting were performed for significant variables with their adjusted odds ratio and 95% confidence interval.

## Results

### Socio-demographic characteristics of participants

In this study, a total of 398 study participants participated with a response rate of 100%, and most of them were aged from 25–34 years with a median (±IQR) age of 30 (±8) years having a minimum and maximum age of 21 and 56 years respectively. The median family size of participants was 3 (±3) having minimum and maximum sizes of 1 and 10, respectively. Besides, Participants ranged in work experience from 1 year to 36 years, with a median of 6 (±6) years (Table 1).

**Table 1:** Socio-demographic characteristics of study participants by the factors associated with COVID-19 vaccine uptake among health professionals in Debre Markos town public health facility, East Gojjam zone, North West Ethiopia (N = 398).

S.No	Variables		Number	Percent
1	Sex	Male	236	59.30
		Female	162	40.70
2	Age	18–24	48	12.06
		25–34	247	62.06
		> = 35	103	25.88
3	Marital status	Single	162	40.7
		Married	221	55.5
		Divorced	11	2.8
		Windowed	4	1.0
4	Religion	Orthodox	349	87.69
		Muslim	36	9.05
		Other	13	3.27
5	Ethnicity	Amhara	384	96.48
		Oromo	9	2.26
		Tigre	4	1.01
		Other	1	0.25
6	Type of profession	Physician	47	11.81
		Nurse	217	54.52
		Midwifery	21	5.28
		Health officer	28	7.04
		Pharmacy	27	6.78
		Laboratory	22	5.53
		Radiology	7	1.76
		Anesthesia	11	2.76
		Other*	18	4.52
7	Income	3201–6193	114	28.64
		6194–9056	151	37.94
		>9056	133	33.42
8	Family Size	< 4	326	81.91
		≥ 4	72	18.09
9	Work Experience	<6	207	52.01
		≥ 6	191	47.99
10	Partner Occupation	Health Worker	72	30.51
		Other Public Servant	76	32.20
		Private employee	27	11.44
		Merchant	31	13.14
		No job	19	8.05
		Other	11	4.66
11	Living condition	Alone	79	25.82
		With partner	59	19.28
		With children	43	14.05
		With a partner and children	111	36.27
		Other	14	4.58

* includes Health informatics technician and Emergency medical technicians

### Health-related characteristics of participants

More than ninety percent of the study participants were given direct patient care. Besides, 227 (57.04%) of participants have been immunized with other vaccines in adulthood. Sixty-three (15.83%) of the health professionals in this study have chronic diseases. One hundred seventy (42.71%) of the respondents have been working in the outpatient department (Table 2).

**Table 2:** Health-related characteristics towards factors associated with COVID-19 vaccine uptake among health professionals in Debre Markos town public Health Facilities, East Gojjam zone, North West Ethiopia (N = 398).

S.No	Variables			Number	Percent
1	Type of Health Facility		Health Center	88	22.21
			Hospital	310	77.89
2	Working Room within the facility		Intensive care Unit (ICU)	17	4.27
			emergency Outpatient room	57	14.32
			Operative room (OR)	25	6.28
			Isolation ward	3	0.75
			Outpatient department (OPD)	170	42.71
			All ward	95	23.87
			none clinical Room	20	5.03
			Other	11	2.76
3	Previous Adulthood other immunization History		Yes	227	57.04
			No	171	42.96
4	Chronic Disease status	Yes	Diabetes Mellitus	19	4.77
			Asthma	30	7.54
			Hypertension	8	2.01
			HIV/AIDS	4	1.01
			Other	2	0.5
		No		335	84.17
5	Direct patient contact		Yes	359	90.20
			No	39	9.80

### COVID-19-related characteristics of participants

Over 90% of the healthcare providers employed at Debre Markos town’s public health facilities were well-versed in COVID-19 and its vaccine. But, more than 35% of health professionals have not practiced the COVID-19 prevention method in this study. Out of the total health professionals who participated in the study, 173 (43.47%) had a history of contact with patients who had been infected by COVID-19 (Table 3).

**Table 3:** COVID-19 related characteristics towards the factors associated with COVID-19 vaccine uptake among health professionals in Debre Markos town public health facilities, East Gojjam zone, North West Ethiopia (N = 398).

S.No	Variables		Number	Percent
**1**	Practicing COVID-19 Prevention methods	Yes	255	64.07
		No	143	35.93
**2**	Wearing Mask	Yes	286	71.86
		No	112	28.14
**3**	Washing Hands/rub	Yes	275	69.10
		No	123	30.90
**4**	Previous PCR + history	Yes	51	12.81
		No	347	87.19
**5**	COVID-19 Patient Contact History	Yes	173	43.47
		No	225	56.53
**6**	Source of information about COVID-19 Disease	Training	56	14.07
		Reading Books	85	21.36
		Social Media	174	43.72
		Mass Media	73	18.34
		Other	10	2.51
**7**	Source of information about the COVID-19 vaccine	Training	52	13.07
		Reading Books	47	11.81
		Social Media	168	42.21
		Mass Media	119	29.90
		Other	12	3.02
**8**	Knowledge about COVID-19	Good	362	90.95
		Poor	36	9.05
**9**	Attitude to the COVID-19 vaccine	Favorable	356	89.45
		Unfavorable	42	10.55

### The magnitude of the uptake of the COVID-19 vaccine

**Fig 1:**
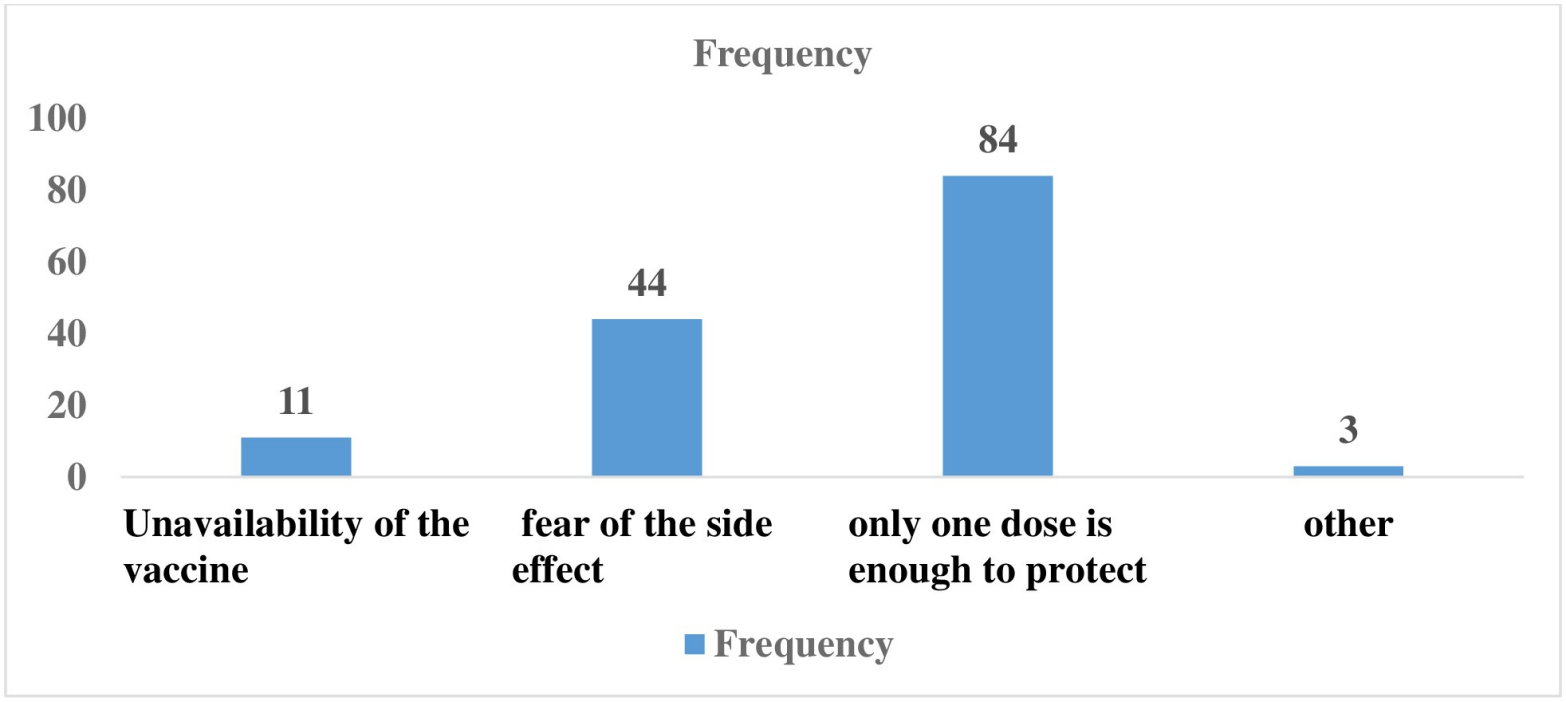
Reasons of Health professionals for not taking the second dose of the COVID-19 vaccine among health professionals working at Debre Markos town public health facilities, East Gojjam zone, North West Ethiopia (N = 398) 2022.

The overall uptake of the COVID-19 vaccine among Health professionals working in Debre Markos town Public Health facilities was 61.56% (245) (95% CI: 56.67%, 66.23%). Among these, 142 (57.96%), 88 (35.92%), and 15(6.12%) were taken with the vaccine in one dose, two-dose, and booster doses respectively. Besides, those who took the first dose vaccine, but not the second dose have the following reasons explained in Fig 1. Similarly, among those who took the vaccine, 73 (29.80%) had no complaints of side effects, 71(28.98%) felt headache 83 (33.88%) explained they had joint pain, 12 (4.90%) had diarrhea and vomiting, and 6 (2.45%) of them had other side effect complaints. Eighty (80) (32.65%) of those who had side effects from the vaccine had taken medication.

**Fig 2:**
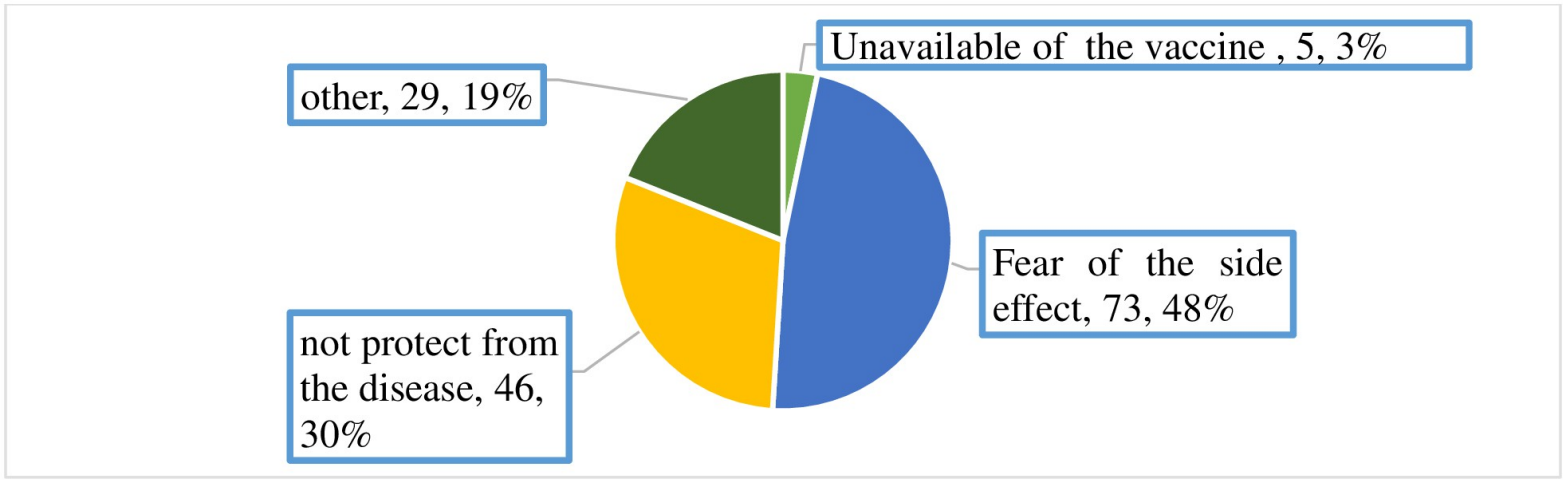
Reasons of health professionals for not taking COVID-19 vaccine among health professionals working at Debre Markos town public health facilities, East Gojjam zone, North West Ethiopia (N = 398)2022.

Of the total participants, 153 (38.44%) had not got the vaccination with at least one dose of the COVID -19 vaccine. Different reasons were given by these respondents. The major reason for not taking the vaccine was fear of side effects (Fig 2).

### Factors associated with the uptake of the COVID-19 vaccine

Statistically significant variables were identified using binary logistic regression modeling and interpreted as follows (Table 4).

**Table 4:** Multivariable logistic regression of factors associated with COVID-19 vaccine uptake among health professionals in Debre Markos town public health facilities, East Gojjam zone, North West Ethiopia (N = 398).

S.No	Variable		Uptake of COVID -19 Vaccine				
			Yes #	No #	COR(95%CI)	AOR(95% CI)	P-value
1	Age	18–24	20	28	1.00	1.00	
		25–34	141	106	1.86 (0.99, 3.48)	1.69 (0.86, 3.33)	0.128
		> = 35	84	19	6.19 (2.89, 13.23)	**4.39 (1.89, 10.19)**	0.001*
2	Income	3201–6193	56	58	1.00	1.00	
		6194–9056	96	55	1.81 (0.91, 2.96)	1.74 (0.96, 3.15)	0.07
		>9056	93	40	2.41 (1.43, 4.07)	**1.79 (1.03, 3.10)**	**0.039** *
3	Practicing COVID-19 Prevention methods	Yes	177	78	2.50 (1.64, 3.82)	**2.39 (1.51, 3.77)**	**<0.001** *
		No	68	75	1.00	1.00	
4	Previous Adulthood other immunization History	Yes	154	73	1.85 (1.23, 2.79)	**1.63 (1.15, 2.56)**	**0.032** *
		No	91	80	1.00	1.00	
5	Chronic Disease status	Yes	48	15	2.24 (1.21, 4.16)	**1.90 (1.07, 3.74)**	**0.043** *
		No	197	138	1.00	1.00	
**6**	Attitude	Favorable	223	133	1.52 (0.80, 2.90)	1.67 (0.81, 3.48)	0.168
		Unfavorable	22	20	1.00	1.00	

^#^ = Expressed in counts/number

* = statistically significant

Those health professionals whose ages were greater than or equal to 35 years were 1.89 (AOR: 4.39, 95% CI: 1.89, 10.19) times more likely to uptake the COVID-19 vaccine than those whose ages were from 18–24 years. Similarly, those who had higher income (>9056 Birr) were 1.79 (AOR: 1.79, 95% CI: 1.03, 3.10) times more likely to take COVID-19 Vaccine than those who had lower income (3201–6193 Birr).

Besides, the odds of uptake of the COVID-19 vaccine among health professionals who practiced COVID-19 prevention methods were 2.39 (AOR: 2.39, 95% CI: 1.51, 3.77) times higher than those who did not practice COVID-19 Prevention method.

Among those health professionals who had Adulthood other immunization histories (AOR: 1.63, 95% CI: 1.15, 2.56) and chronic disease (AOR: 1.90, 95% CI: 1.07, 3.74) were more likely up taking COVID-19 vaccine than those who had no history of adulthood immunization and chronic disease respectively (Table 4).

## Discussion

Vaccines are life-saving interventions that have been responsible for the elimination and control of many infectious diseases in many parts of the world. It has been demonstrated that vaccinations not only offer direct immunity and shield vaccinated individuals from the disease, but also, if a larger percentage of the community is immune, guarantee people who have not been immunized through herd immunity. [1]. Uptake of the COVID-19 vaccine among healthcare workers is mandatory to minimize and reduce the chain of transmission of COVID-19; and is an important challenge to be addressed [31]. So this study revealed the magnitude of uptake of the COVID-19 vaccine and associated factors among health professionals who have worked at Debre Markos Town public health facilities.

The uptake of the COVID-19 vaccine among Health professionals who have worked at Debre Markos town public Health facilities was 61.56% (95% CI: 56.67, 66.23). This result is in alignment with the results of the Ethiopian study, which stated that 62.1% of health care professionals had taken the Vaccine [27, 34]. But this finding is higher than the study done in Saudi Arabian countries that revealed 33.27% of Vaccine Uptake [16]. This difference is possibly because of the socio-demographic difference between the two countries. Besides, the finding of this study was lower than the finding of the Californian, and the USA study [13, 14]. It possibly because of the difference in morbidity and mortality rates of the disease in these countries, which indirectly affect the vaccine uptake. In USA and California, morbidity and mortality caused by COVID-19 were very high than in Ethiopia [13, 14]. In addition, the finding of our study were lower than the findings of the study done in Addis Ababa, Ethiopia [28]. It could be because we conducted our study in January and February of 2022, when there had been a campaign in place and not every healthcare professional had woken up and consented to receive the vaccine. However, nearly all medical professionals who were willing to receive the vaccine did so after the campaign finished. That is why the Addis Ababa study findings were higher than the findings of this study. Similarly, the findings of the study done in Somalia, which reported that 37.4% of health care professionals participated in the study took the vaccine, was lower than the findings of our study [24]. The reason for this discrepancy might be our study was done in a single town level health facility using self-administered questioning whereas the Somalian study was done at a national level which included different states using an online survey. Similarly, the findings of our study were lower than the findings of the study done in Malawi, 80% uptake [35]. The reason behind this may be the Malawi study’s selected study participants’ health institutions purposely (HIV care centers, hospitals and larger health centers); so that the Health professionals uptake level will be varied as the patient contact level varied [10, 36]. Besides, this finding is lower than the wales’ study findings [37]. The reason could be our study selected participants directly from the health institution where they were working, but the Wales study retrieved secondary data from E-cohort of health care workers in Wales. This may affect the level of COVID-19 vaccine uptake.

This study identified that the major reason for not taking the COVID-19 vaccine was fear of side effects (73/153, 48%). This result is in alignment with the results of the study done in Australia and Somalia, which stated that fear of the vaccine’s needle and adverse effects were the reasons that made them less uptake the vaccine [24, 38]. Whereas this finding is not in line with the findings of the study done in the USA, which stated that the major cause of low uptake of the vaccine was hesitancy but not fear of side effects [39]. The finding of this study implies that 18.34% of the sample population is expected to fear the side effect of the COVID- 19 vaccine.

Those health Professionals whose ages were greater than or equal to 35 years were 4.39 (95% CI: 1.89, 10.19) times more likely to uptake the COVID-19 vaccine than those whose ages were from 18–24 years. This result is in alignment with the results of the study done in Ethiopia, Somalia, the USA, and Saudi Arabia which revealed that as age increases, the uptake of the vaccine was also increased [24, 27, 39–44]. This might be due to as the age increases, the risk of mortality and morbidity also increases. So those older health professionals could uptake the COVID-19 vaccine more than the younger to decrease mortality and morbidity in addition to the age-related risk.

Those who had higher income (>9056 Birr) were 1.79 (95% CI: 1.03, 3.10) times more likely to take COVID-19 Vaccine than those who had lower income (3201–6193 Birr). This result is in alignment with the results of a study done in low and middle-income countries, which identified that as socioeconomic status increases, the vaccine acceptance rate also increases [45]. It is because as the living standards of an individual are better, being willing to live longer is expected. But this finding is opposite to the study’s findings done at the international level, which revealed that lower-income societies had low acceptance and uptake rates of the COVID-19 vaccine than higher-income ones [46].

Besides, those health professionals who practiced COVID-19 Prevention method were 2.39 (95% CI: 1.51, 3.77) times higher COVID-19 vaccine uptake than those who did not practice COVID-19 Prevention methods. It is scientifically sound that as the health professional practices prevention methods, the ability of habits to practice more prevention methods is evidenced.

Those health professionals who have an adulthood immunization history were 1.63 (95% CI: 1.15, 2.56) times more likely to uptake the COVID-19 vaccine than those who had no history of immunization in adulthood. This result is in alignment with the results of the study done in Ethiopia, which explained that those who had a previous vaccination history had more likely to uptake the COVID -19 vaccine than those who had no vaccination history in adulthood [27]. It is because the two studies are done at the health professional level, which might have a similar knowledge and practice level of vaccination.

The odds of uptake COVID-19 vaccine among Health professionals who had chronic diseases were 1.9 (95% CI: 1.07, 3.74) times more likely than those who have no chronic disease. These professionals take more concerned to maintain their health and take the vaccine eagerly to increase their ability to protect themselves from COVID-19 disease. But previous studies in China revealed that chronic disease status and uptake of the COVID-19 vaccine uptake had no association [47]. This might be what they thought as the side effect of the vaccine is worse in a chronically ill patient.

### Limitations of the study

The data were collected through self-administered questionnaires, which might have not properly completed questionnaire due to work overload. Besides, data collection was performed during January and February where COVID-19 vaccination was on the campaign, which might increase the magnitude of COVID-19 vaccine uptake among health professionals.

## Conclusion

The uptake of the COVID-19 vaccine among health professionals working in Debre Markos town Public Health facilities was low. The target uptake of the Covid-19 vaccine must be 90% and above. The reasons explained by healthcare professionals for not taking the COVID-19 vaccine were fear of side effects, thinking of not protecting from the disease, and unavailability of the vaccine.

Statistically significant factors for this uptake of the COVID-19 vaccine were age > = 35 years, having a higher income, having adulthood immunization history, practicing COVID-19 prevention methods, and having a chronic disease.

Health professionals should strengthen their evidence about COVID-19 vaccine side effects. It is also better to have sufficient evidence as reason for not taking the vaccine as health professionals are frontline in such cases for the community. Besides, it is better to see these factors further in follow-up study design and qualitative research. In addition to this, the researchers are better to study further by including non-health professionals who are working in the health facility. Generally, policymakers and health managers should think about the requirement of immunization of healthcare workers and develop plans for administering the COVID-19 vaccine.

## Supporting information

S1 Data Anonymized data set. (DTA)
